# Supplementary material for: Structural Basis for the Inhibition of Coronaviral Main Proteases by a Benzothiazole-Based Inhibitor
Source: Viruses. 2022 Sep 18;14(9):2075. doi: 10.3390/v14092075 (PMC9505605; doi:10.3390/v14092075)
Supplement: Supplementary file 1 [file viruses-14-02075-s001.zip › viruses-1891424-supplementary.pdf]

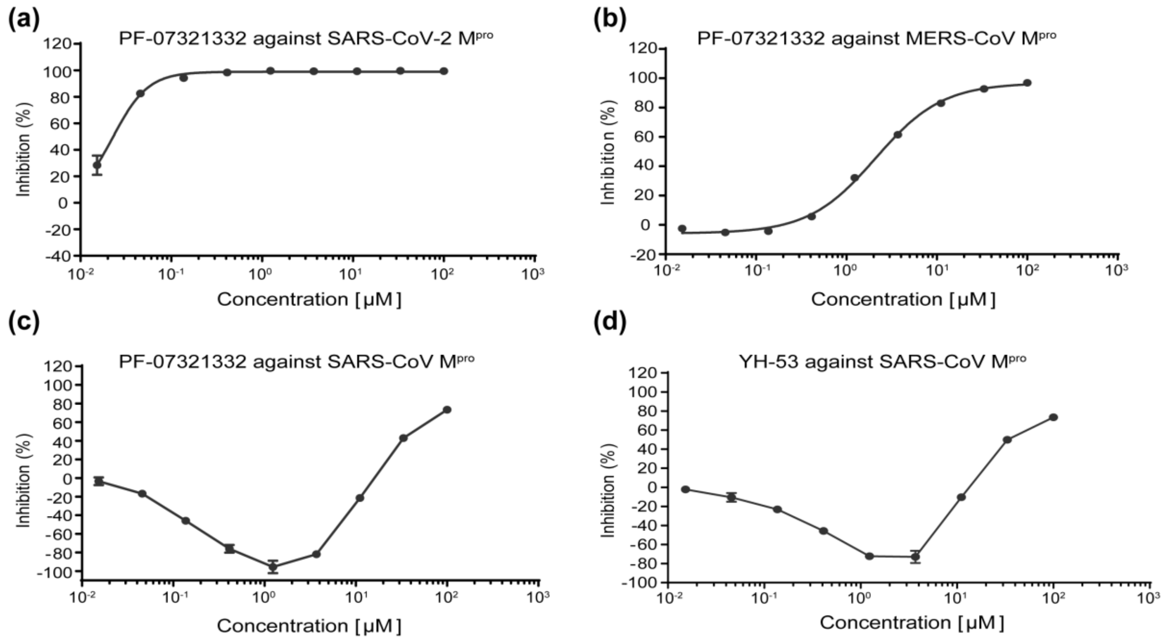

Figure S1. Enzymatic inhibition activities of YH-53 and PF-07321332. (a) Inhibition of PF-07321332 against main protease of SARS-CoV-2. (b) Inhibition of PF-07321332 against main protease of MERS-CoV. (c) Inhibition of PF-07321332 against main protease of SARS-CoV. (d) Inhibition of YH-53 against main protease of SARS-CoV. Main proteases were preincubated in the reaction buffer with various concentrations of YH-53 or PF-07321332 at room temperature for 30 minutes before reacting with the FRET substrate.

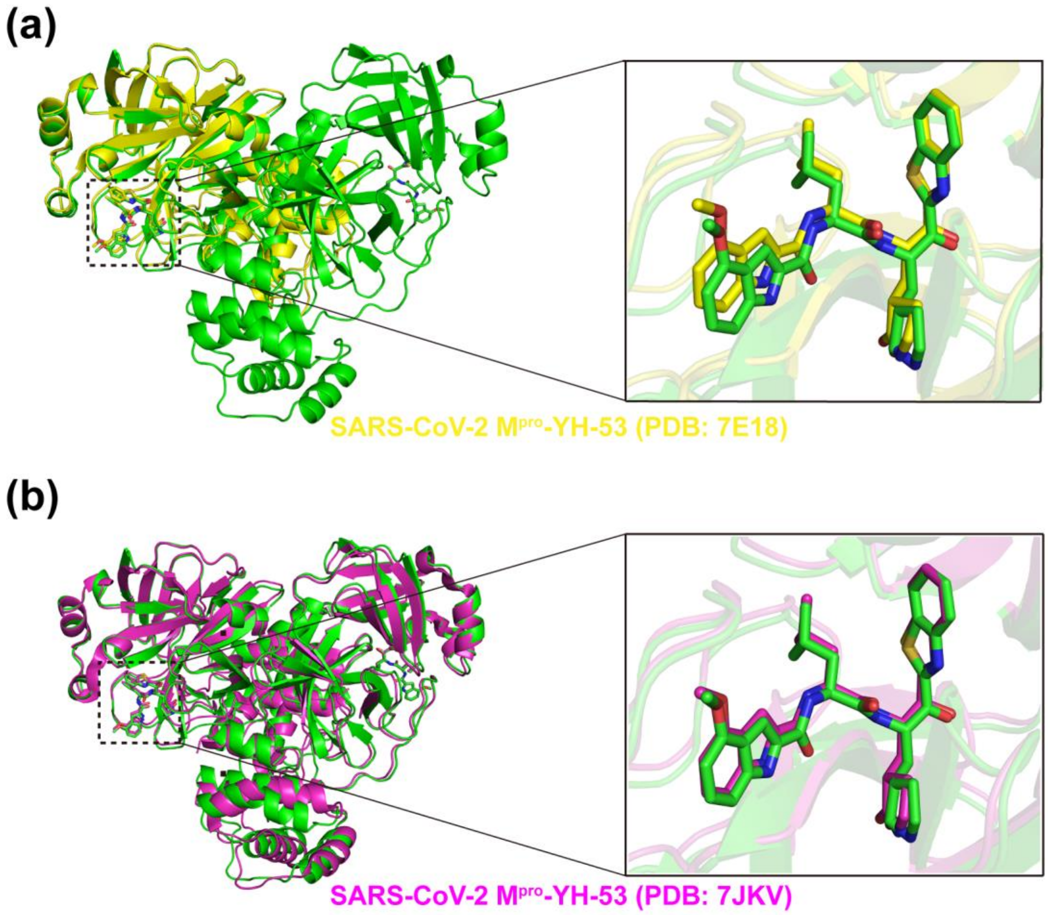

Figure S2. Comparison of the SARS-CoV-2 Mpro-YH-53 structure we reported in this study to SARS-CoV-2 Mpro-YH-53 structures reported previously. **(a)** Comparison of our structure (green) to the SARS-CoV-2 Mpro-YH53 structure (PDB ID 7E18, yellow). **(b)** Comparison of our structure (green) to the SARS-CoV-2 Mpro-YH53 structure (PDB ID 7JKV, magentas).

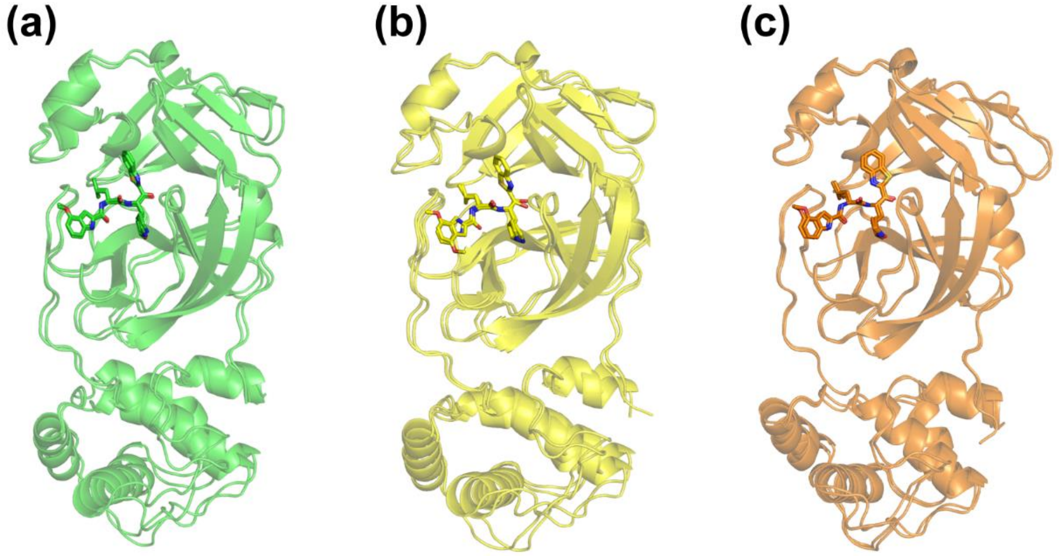

Figure S3. Structural comparison of protomer A and B of SARS-CoV-2, SARS-CoV and MERS-CoV Mpros bound to YH-53. Structural comparison of protomer A and protomer B of SARS-CoV-2 Mpro **(a)**, SARS-CoV Mpro **(b)** and MERS-CoV Mpro **(c)** bound to YH-53. Protomers are shown as cartoons, while ligands are shown as sticks.

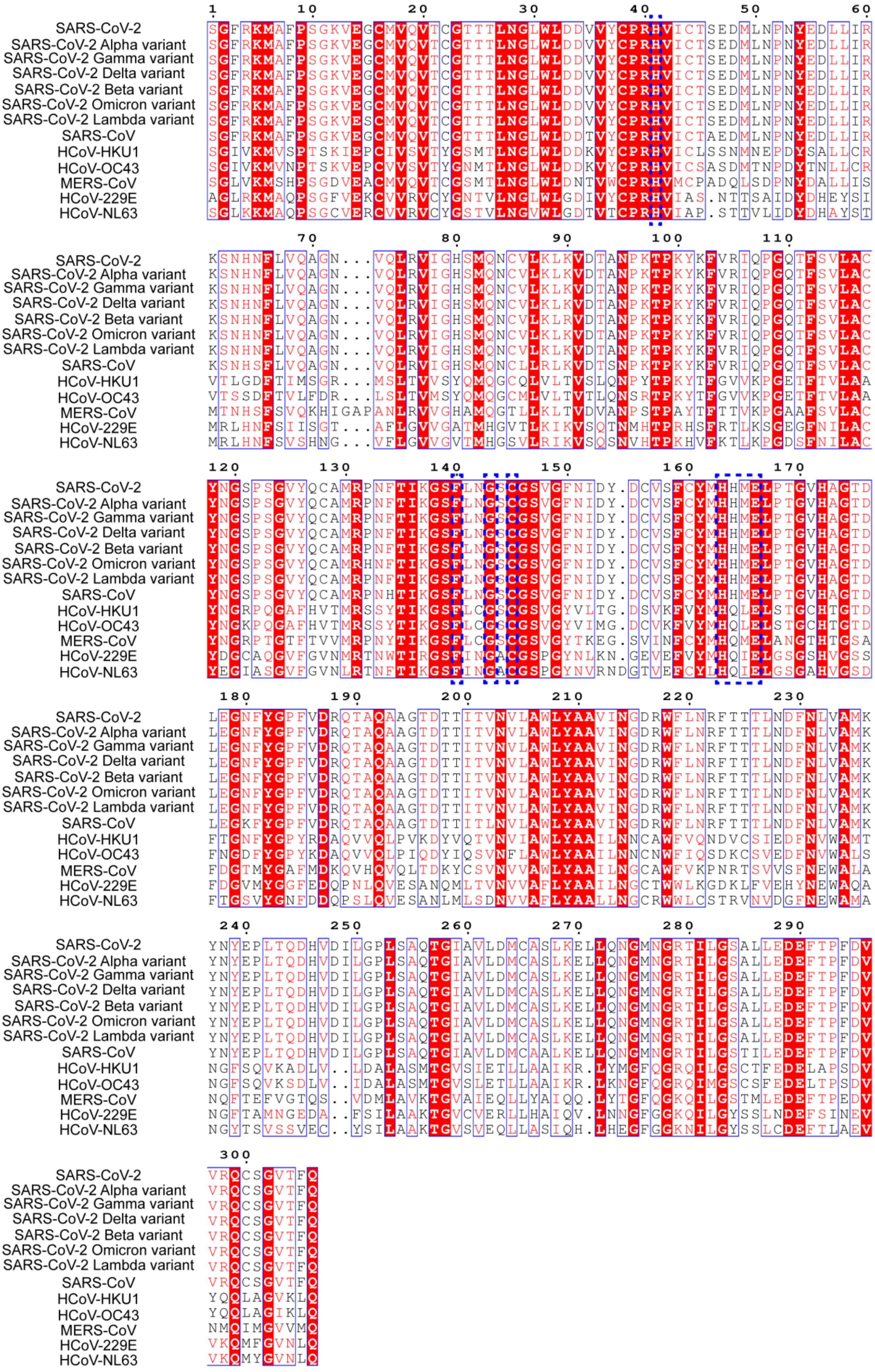
SARS-CoV-2
SARS-CoV-2 Alpha variant
SARS-CoV-2 Gamma variant
SARS-CoV-2 Delta variant
SARS-CoV-2 Beta variant
SARS-CoV-2 Omicron variant
SARS-CoV-2 Lambda variant
SARS-CoV
HCoV-HKU1
HCoV-OC43
MERS-CoV
HCoV-229E
HCoV-NL63

Figure S4. Sequence alignment of M^{pro}s from different coronaviruses. The M^{pro} amino acid sequences of SARS-CoV-2, SARS-CoV, MERS-CoV, HCoV-NL63, HCoV-229E, HCoV-HKU1, HCoV-OC43, SARS-CoV-2 Alpha variant, SARS-CoV-2 Beta variant, SARS-CoV-2 Gamma variant, SARS-CoV-2 Delta variant, SARS-CoV-2 Lambda variant, and SARS-CoV-2 Omicron variant were retrieved from Genbank or GISAID. ClustalX (1.83) software was used for sequence alignment and ESPript 3.0 was used to generate a graphical representation. The key residues involved in interacting with inhibitors were labeled with blue dashed boxes.
